# Supplementary material for: Predictors of Treatment Requirements in Women with Gestational Diabetes: A Retrospective Analysis
Source: J Clin Med. 2021 Sep 27;10(19):4421. doi: 10.3390/jcm10194421 (PMC8509276; doi:10.3390/jcm10194421)
Supplement: Supplementary file 1 [file jcm-10-04421-s001.zip › jcm-1336313-supplementary.pdf]

Journal of
Clinical Medicine

MDPI

Table S1 Perinatal outcome parameters of the different treatment groups

Variable	Data available (N)	Diet control	Insulin	*p*†	Bolus	Basal	MDI	*p*‡
Preterm delivery	328	7.4%	7.9%	1	12.5%	5.1%	6.7%	.618
C-Section	332	28%	37.1%	.096	33.3%	37.5%	39.3%	.325
LGA	322	12.8%	14.1%	.743	5.1%	15.8%	19%	.251
SGA	322	7.5%	3.7%	.230	5.1%	2.6%	3.4%	.522
NICU	303	12.1%	11.6%	1	16.1%	10.5%	9.4%	.793
Hyperbilirubinemia	225	24.4%	30%	.361	21.7%	40%	27%	.340
Hypoglycemia	202	3.5%	7.1%	.167	4.5%	10.7%	5.9%	.289

*significant after Bonferroni correction for multiple testing ($p<.05$); † Comparing diet vs. insulin groups ‡ comparing diet, bolus, basal and MDI (multiple daily injections); LGA, large for gestational age (birthweight >90th percentile); NICU, neonatal intensive care unit; SGA, small for gestational age (birthweight <10th percentile). The percentages shown in the subgroups represent the frequency of the variables relative to the number of cases in the first column.
